# Associations of vitamin D, hepatic function and inflammatory biomarkers with obesity: A cross-sectional analysis

**DOI:** 10.6026/973206300223469

**Published:** 2026-06-30

**Authors:** Habibatou Diabate, Clifton Mason Perry, Sajidha Nizamudeen, Oluwatobi Onalaja, Sabah Fatima, Ram Vikas Reddy, Mohammed Hady Albitar, Abdullah Almahmoud, Alana Rivera Negrón, Harsh Vijay Sanghvi, Mohammed Abdul Mateen

**Affiliations:** 1 Department of Internal Medicine, American University of the Caribbean school of medicine, Sint Maarten; 2 Department of Internal Medicine, Ross University School of Medicine, Miramar, Florida, USA; 3 Department of Internal Medicine, Travancore Medical College, Kerala, India; 4 Department of Internal Medicine, KBN University, Khaja Banday Nawaz medical college, Kalaburagi, Karnataka, India; 5 Department of Internal Medicine, NRI Medical College, Guntur, India; 6 Department of Internal Medicine, College of Medicine, Alfaisal University, Riyadh, Saudi Arabia; 7 Department of Medicine, College of Medicine, Alfaisal University, Riyadh, Saudi Arabia; 8 Department of Internal Medicine, University of Medicine and Health Sciences, Basseterre, St. Kitts & Nevis; 9 Department of Internal Medicine, Georgian National University SEU, Tblisi, Georgia; 10 Department of Anaesthesia, Mahavir Institute of Medical Sciences, Vikarabad, India

**Keywords:** Obesity, vitamin D, liver enzymes, inflammatory biomarkers, non-alcoholic fatty liver disease (NAFLD)

## Abstract

Obesity is associated with hepatic dysfunction, chronic inflammation and vitamin D deficiency, but their interrelationship remains incompletely understood. Therefore, it is of interest to compare the liver function, inflammatory and vitamin D-PTH biomarkers between 100 obese adults (BMI ≥30 kg/m^2^) and 100 age-matched non-obese controls. Obese participants showed significantly higher ALT, AST, ALP, GGT, CRP, IL-6, fibrinogen and PTH levels, with lower serum 25(OH)D concentrations. Regression analysis identified CRP (OR=1.38, 95% CI: 1.24-1.54) and GGT (OR=1.35, 95% CI: 1.20-1.52) as the strongest positive predictors, while 25(OH)D showed a significant inverse association with obesity (OR=0.79, 95% CI: 0.68-0.91). Thus, data shows the combined contribution of hepatic dysfunction, systemic inflammation and vitamin D imbalance to obesity-related metabolic disturbances and support their early clinical assessment.

## Background:

Obesity is a leading cause of non-communicable diseases (NCDs) and remains a major global health challenge. Rapid urbanization, dietary changes and lifestyle shifts are key contributors to the rising prevalence of adult obesity [1]. According to NFHS-5 (2019-2021), 24% of females and 22.9% of males have a body mass index (BMI) ≥ 25 kg/m^2^, with a higher prevalence in metropolitan areas. Obesity has a complex etiology, influenced by low physical activity, stress, genetic susceptibility, endocrine dysfunction, sedentary behavior and diets rich in processed and high-calorie foods. Obesity not only increases the risk of cardiovascular and metabolic disorders, but also has systemic effects, including hepatic impairment and altered immune responses [2]. Liver function tests (LFTs) are commonly utilized to assess hepatic function and detect early increased risk of developing hepatic steatosis, suggesting a potential role in the pathogenesis of NAFLD [6]. Therefore, analyzing the relationship between vitamin D deficiency, liver function and inflammatory markers in obese individuals is crucial for identifying early signs of metabolic dysregulation and guiding therapeutic approaches. While previous studies have independently examined the associations between obesity, liver function, inflammation and vitamin D status, limited data are available integrating these domains within a single analytical framework. Therefore, it is of interest to evaluate liver function tests, inflammatory markers and vitamin D-PTH status in obese individuals and determine their interrelationship.

## Methods:

## Study design and population:

This was a cross-sectional analytical study with a comparative design including obese and non-obese groups. The study was conducted in the Department of General Medicine and Clinical Biochemistry at a tertiary care teaching hospital in India. Data collection was carried out over a one-year period, from February 2024 to March 2025, among adults aged 25-55 years. This study was reported in accordance with the STROBE (Strengthening the Reporting of Observational Studies in Epidemiology) guidelines.

## Sample size and sampling:

The sample size was estimated using G*Power software (version 3.1.9.7) for detecting differences between two independent groups, as the primary objective was to compare biomarker levels between obese and non-obese participants. Assuming a moderate standardized effect size (Cohen's d = 0.5), 80% statistical power and a significance level of 5%, a minimum sample size of 84 participants was required. To account for possible dropouts or incomplete data, 100 obese participants were enrolled and an age-matched control group of 100 non-obese individuals was also included.

## Inclusion and exclusion criteria:

Participants in this study were adults aged 25 to 55 years classified as obese (BMI ≥30 kg/m^2^) according to World Health Organization (WHO) criteria [7]. In addition, an age-matched control group of 100 non-obese individuals (BMI <25 kg/m^2^) was included for comparative analysis. Only participants who provided informed consent and underwent blood testing were included. Those with chronic liver diseases (e.g. hepatitis B/C, autoimmune hepatitis, cirrhosis), alcohol intake exceeding 10 g/day in females or 20 g/day in males, recent vitamin D supplementation within the past 3 months, endocrine disorders (hypothyroidism, Cushing's syndrome), malignancy, renal impairment, or acute infections were excluded. Additionally, lactating and pregnant women were excluded [8].

## Data collection:

Participants were enrolled consecutively from outpatient clinics and routine health check-up visits at a tertiary care teaching hospital. Recruitment was limited to individuals undergoing general medical evaluation. Both obese and non-obese participants were selected from the same clinical setting to ensure comparability between groups.

## Socio-demographic characteristics:

Clinical and lifestyle data, such as sun exposure, dietary habits, physical activity and medication use, were collected using a structured questionnaire. The height and weight of each participant were recorded according to standardized protocols and BMI was calculated with the following formula: weight (kg)/height (m^2^) [9].

## Blood sample collection and laboratory analysis:

A 10 mL fasting venous blood sample was collected after an 8-10 hour fast. Samples were centrifuged immediately and divided into aliquots for biochemical analysis. 25(OH)D levels were measured by chemiluminescence immunoassay (CLIA) and classified into deficient (<20 ng/mL), insufficient (20-29 ng/mL), or sufficient (≥30 ng/mL). Bioavailable and free vitamin D were calculated and vitamin D-binding protein (DBP), calcium and parathyroid hormone (PTH) levels were measured using standard automated methods [10]. Liver function tests were performed using an automated biochemistry analyzer and included ALT, AST, ALP, GGT and total and direct bilirubin (DBIL). High-sensitivity C-reactive protein (hs-CRP) was measured using an immunoturbidimetric assay, while cytokines including interleukin-6 (IL-6), tumor necrosis factor-alpha (TNF-α) and interleukin-1β (IL-1β) were measured using ELISA kits. Fibrinogen was estimated by the Clauss method and erythrocyte sedimentation rate (ESR) by the Westergren method [11].

## Data analysis:

All quantitative variables were summarized using descriptive statistics and expressed as mean ± standard deviation (SD). Distribution normality was tested using the Kolmogorov-Smirnov test. Intergroup differences between obese and non-obese participants were analyzed using independent t-tests for continuous variables and chi-square tests for categorical data. The level of statistical significance was predefined at α = 0.05 and p-values < 0.05 were interpreted as statistically significant. P-values for group comparisons were calculated using independent t-tests. To evaluate the independent association between biomarkers and obesity status, multivariable logistic regression analysis was performed. Obesity status (obese = 1, non-obese = 0) was used as the dependent variable and the studied biomarkers as independent variables. Adjusted odds ratios (OR) with 95% confidence intervals (CI) were reported. The models were adjusted for potential confounders including age, sex, physical activity level, dietary habits, smoking status, alcohol intake and relevant clinical conditions such as diabetes and hypertension. Multicollinearity among independent variables was assessed using the variance inflation factor (VIF).

## Ethical approval:

This study was conducted in accordance with the Declaration of Helsinki and ethical guidelines for studies that involve human subjects. Ethical approval was obtained from the Institutional Ethics Committee with the IRB number HIMS/IRB2023-24/10874/14A. Prior to data collection, written informed consent was obtained from all participants after informing them about the study's objectives, procedures and confidentiality of their information. Participants were also informed that they could withdraw from the study at any point.

## Results:

The baseline demographic and clinical characteristics of the participants are presented in Table 1. A total of 200 participants were included, comprising 100 non-obese and 100 obese individuals. There was no statistically significant difference between the two groups with respect to age (39.8 ± 8.2 vs. 40.5 ± 7.9 years; p = 0.56) and sex distribution (male/female: 54/46 vs. 57/43; p = 0.68), indicating that the groups were comparable at baseline. As expected, body mass index (BMI) was significantly higher in the obese group compared to the non-obese group (32.6 ± 2.7 vs. 23.4 ± 1.8 kg/m^2^; p < 0.001). Considering lifestyle factors, no significant differences were observed in smoking (18% vs. 22%; p = 0.47) and alcohol consumption (12% vs. 15%; p = 0.52) between the groups. However, a significantly lower proportion of obese individuals reported being physically active compared to non-obese participants (41% vs. 64%; p = 0.003). In terms of clinical comorbidities, the prevalence of diabetes (28% vs. 14%; p = 0.01) and hypertension (34% vs. 16%; p = 0.002) was significantly higher in the obese group. The comparative analysis of liver function biomarkers between obese and non-obese participants is presented in Table 2. ALT levels were significantly higher in the obese group compared to the non-obese group (32.34 ± 11.55 vs. 21.32 ± 7.67 U/L; p = 0.001). Similarly, AST levels were higher in obese participants (27.83 ± 9.13 vs. 20.58 ± 6.41 U/L; p = 0.020). Alkaline phosphatase (ALP) also showed a significant increase in the obese group (102.38 ± 20.06 vs. 78.02 ± 16.80 U/L; p = 0.001). Gamma-glutamyl transferase (GGT) showed the largest increase among obese individuals (43.37 ± 13.33 vs. 25.62 ± 8.93 U/L; p < 0.001). Prealbumin levels were slightly but significantly higher in obese participants (28.25 ± 4.30 vs. 26.67 ± 3.46 mg/dL; p = 0.008). In contrast, serum albumin levels were significantly lower in the obese group (3.91 ± 0.66 vs. 4.06 ± 0.47 g/dL; p < 0.001), indicating possible impairment in liver synthetic function. Total bilirubin (7.23 ± 3.45 vs. 9.67 ± 2.67 µmol/L; p < 0.001) and direct bilirubin (3.00 ± 1.11 vs. 3.45 ± 1.08 µmol/L; p < 0.001) were also lower in the obese group. Table 3 shows the differences in inflammatory biomarkers between the two groups. Systemic inflammation was significantly more pronounced among obese individuals. CRP levels were markedly elevated in obese participants (9.33 vs. 2.44 mg/L; p < 0.001). IL-6 levels were also higher in the obese group (8.10 vs. 5.07 pg/mL; p = 0.044) and TNF-α levels were more than twofold higher in obese participants (12.30 vs. 5.36 pg/mL; p = 0.040). Fibrinogen levels were significantly increased in obese individuals (3.39 vs. 2.66 g/L; p = 0.010). Although IL-1β (1.06 vs. 0.75 pg/mL; p = 0.144) and ESR (14.8 vs. 11.3 mm/hr; p = 0.090) tended to be higher in obese individuals, these differences did not reach statistical significance, indicating that not all inflammatory mediators were uniformly affected. CRP = C-reactive protein; IL-6 = interleukin-6; TNF-α = tumor necrosis factor-alpha; IL-1β = interleukin-1 beta; ESR = erythrocyte sedimentation rate. P < 0.05 was considered statistically significant. P-values were calculated using independent t-tests. Vitamin D-related parameters showed mixed results between obese and non-obese groups (Table 4). Serum 25(OH)D levels were slightly lower in obese participants (26.71 vs. 27.17 ng/mL; p = 0.030). In contrast, bioavailable and free vitamin D levels were significantly higher in obese participants (4.88 vs. 4.79 ng/mL; p = 0.003 and 15.79 vs. 15.04 pg/mL; p = 0.011, respectively). Vitamin D-binding protein (DBP) levels demonstrated higher values in the obese group (413 vs. 338 mg/L), although the difference was not statistically significant (p = 0.231). Parathyroid hormone (PTH) levels were also elevated in obese participants (49.8 vs. 38.5 pg/mL; p = 0.010), while total serum calcium did not differ significantly between the groups despite higher mean values in obese participants (p = 0.300). The multivariable logistic regression analysis of liver function biomarkers is presented in Table 5. Among studied parameters, GGT showed the strongest positive association with obesity (OR = 1.35, 95% CI: 1.20-1.52, p < 0.001), indicating higher odds of obesity with increasing GGT levels. For ALT (OR = 1.22, 95% CI: 1.10-1.36, p = 0.001), ALP (OR = 1.24, 95% CI: 1.11-1.38, p = 0.001) and AST (OR = 1.18, 95% CI: 1.02-1.32, p = 0.020), all were significantly associated with obesity. In contrast, serum albumin (OR = 0.82, 95% CI: 0.71-0.94, p < 0.001) and prealbumin (OR = 0.85, 95% CI: 0.76-0.96, p = 0.008) showed significant inverse associations with obesity, suggesting that lower levels of these proteins are associated with increased likelihood of obesity. TBIL (OR = 0.94, 95% CI: 0.82-1.07, p = 0.31) and DBIL (OR = 0.91, 95% CI: 0.79-1.05, p = 0.22) demonstrated inverse but statistically non-significant associations with obesity. Table 6 shows the multivariable logistic regression analysis of inflammatory biomarkers. Among the studied parameters, CRP demonstrated the strongest positive association with obesity (OR = 1.38, 95% CI: 1.24-1.54, p < 0.001). IL-6 was also significantly associated with obesity (OR = 1.21, 95% CI: 1.05-1.38, p = 0.044). Fibrinogen showed a significant positive association as well (OR = 1.19, 95% CI: 1.07-1.32, p = 0.010), further supporting the presence of a pro-inflammatory and pro-thrombotic state in obese individuals. In contrast, TNF-α (OR = 1.09, 95% CI: 0.95-1.25, p = 0.18), interleukin-1 beta (IL-1β) (OR = 1.12, 95% CI: 0.97-1.29, p = 0.14) and erythrocyte sedimentation rate (ESR) (OR = 1.08, 95% CI: 0.96- 1.21, p = 0.09) demonstrated positive but statistically non-significant associations with obesity. The multivariable logistic regression analysis of vitamin D-related parameters and calcium is presented in Table 7. 25(OH)D levels had a significant inverse association with obesity (OR = 0.79, 95% CI: 0.68-0.91, p = 0.030), indicating that higher vitamin D levels are associated with lower odds of obesity. Similarly, bioavailable vitamin D (OR = 0.83, 95% CI: 0.72-0.95, p = 0.003) and free vitamin D (OR = 0.85, 95% CI: 0.74-0.97, p = 0.011) were also significantly inversely associated with obesity. In contrast, parathyroid hormone (PTH) showed a significant positive association with obesity (OR = 1.18, 95% CI: 1.06-1.31, p = 0.010), suggesting an increased likelihood of elevated PTH levels among obese individuals. Vitamin D-binding protein (DBP) demonstrated a positive but statistically non-significant association (OR = 1.08, 95% CI: 0.95-1.22, p = 0.23). Similarly, serum calcium showed a non-significant inverse association with obesity (OR = 0.95, 95% CI: 0.81-1.11, p = 0.30). Although mean calcium levels appeared higher in obese participants, this difference was not statistically significant (p = 0.300), which may be attributed to variability within the groups.

## Discussion:

This study evaluated the associations between vitamin D-related parameters, liver function markers and inflammatory biomarkers in obese individuals, with comparative analysis against non-obese individuals. Our findings indicate that obesity is associated with significant alterations in liver enzyme activity, inflammatory status and certain vitamin D-related parameters, consistent with previous reports [12, 13, 14]. Individuals with obesity had higher levels of AST, ALT, ALP and GGT than non-obese participants and these associations remained significant in regression analyses, with GGT showing the strongest positive associations with obesity. These findings suggest that liver enzyme elevations, particularly GGT, may reflect early hepatic dysfunction associated with obesity. Elevated liver enzymes in obesity are largely attributed to NAFLD, in which excessive caloric intake and adiposity lead to lipid accumulation within hepatocytes. This process induces oxidative stress, mitochondrial dysfunction and hepatocellular injury, leading to enzyme leakage into the circulation [15]. Elevated ALP and GGT levels may also indicate subclinical cholestasis or biliary epithelial stress. Numerous studies have highlighted the antioxidant and anti-inflammatory properties of bilirubin; therefore, reduced bilirubin levels may weaken the antioxidant effect and predispose individuals to metabolic dysfunction [16]. The inverse association between serum albumin and obesity observed in this study supports prior evidence that chronic low-grade inflammation suppresses hepatic synthetic function through cytokine-mediated downregulation of albumin gene transcription. Prealbumin levels were slightly higher in obese individuals, possibly reflecting preserved short-term hepatic synthetic capacity or nutritional status rather than inflammatory suppression [17]. CRP and IL-6 levels were markedly elevated in obese participants, with both showing significant positive associations with obesity in regression models. Visceral adipose tissue may act as an endocrine organ, releasing pro-inflammatory adipokines such as IL-6 and TNF-α. IL-6 promotes hepatic synthesis of acute-phase reactants, such as CRP and fibrinogen, thereby linking adipose inflammation to impaired liver function [18]. Elevated TNF-α may also contribute to insulin resistance through disruption of insulin receptor signaling, whereas elevated fibrinogen levels may suggest activation of pro-coagulant pathways, predisposing obese individuals to increased cardiovascular risk. Although IL-1β and ESR were higher in the obese group, these differences were not statistically significant, which is consistent with reports showing variable associations between these markers and obesity [19]. A reduction in serum 25(OH)D levels was observed in obese individuals, supporting previous epidemiological studies indicating an inverse relationship between vitamin D status and body mass index. Although statistically significant, the absolute difference in 25(OH)D levels between groups was small and may not be clinically meaningful. Notably, both groups exhibited mean vitamin D levels within the insufficient range, indicating a high prevalence of suboptimal vitamin D status irrespective of obesity. Since vitamin D is fat-soluble, it can become sequestered in adipose tissue, which diminishes its bioavailability [20]. Chronic inflammation may contribute to impaired vitamin D activation in the liver and kidneys through cytokine-mediated suppression of hydroxylase enzymes. In contrast to most reports, bioavailable and free vitamin D showed slightly higher mean values in the obese group. However, in multivariable regression analysis, both bioavailable and free vitamin D demonstrated inverse associations with obesity. This apparent discrepancy may reflect confounding effects, assay variability, altered DBP dynamics, or population-specific differences and should therefore be interpreted with caution. Elevated PTH levels in obesity likely represent secondary hyperparathyroidism, a compensatory response that maintains calcium homeostasis when vitamin D bioavailability is reduced [21]. DBP and calcium levels did not differ significantly between groups, suggesting that these parameters may be less sensitive indicators of adiposity-related changes.

## Strengths and Limitations:

Our findings reinforce the concept of obesity as a chronic state of low-grade inflammation and metabolic dysfunction, proposed by Goldstone in 2022 and subsequently supported by large population-based studies. Excess adiposity drives hepatic lipid accumulation and low- grade inflammation, impairing liver function and altering vitamin D metabolism. The strong associations observed for CRP, IL-6 and GGT highlight their potential use as biomarkers for cardiometabolic risk assessment in clinical practice. Furthermore, the inverse relationship between 25(OH)D and obesity is consistent with evidence from meta-analyses, suggesting that vitamin D supplementation could contribute to weight management strategies, although a causal link has not yet been established. Additionally, the findings support the potential role of routine screening of liver enzymes and inflammatory markers in obese individuals for early identification of metabolic risk. It is difficult to draw conclusions regarding causal relationships due to the study's cross-sectional design. Additionally, potential confounders, including dietary intake, physical activity, sunlight exposure and advanced hepatic imaging, were not accounted for, which may limit the generalizability of the findings to other populations. Moreover, potential multicollinearity among liver enzymes included in the regression models may have influenced the strength of the observed associations. The relatively small sample size may also limit the statistical power to detect weaker associations. Further research is required to determine whether correcting vitamin D deficiency, reducing inflammation and improving hepatic function could improve metabolic outcomes in obese individuals. Prospective longitudinal studies are needed to clarify the temporal relationships between vitamin D deficiency, systemic inflammation and liver dysfunction in obese individuals.

## Conclusion: 

Obesity was characterized by hepatic injury, persistent low-grade inflammation and disruption of the vitamin D-PTH axis. The coexistence of these abnormalities highlights their potential role in driving obesity-related metabolic complications. Thus, integrated monitoring of liver function, inflammatory burden and vitamin D status may facilitate earlier risk stratification and intervention.

## Transparency statement:

The corresponding author, Raghabendra Kumar Mahato, affirms that this manuscript is an honest, accurate and transparent account of the study being reported; that no important aspects of the study have been omitted; and that any discrepancies from the study as planned (and, if relevant, registered) have been explained.

## Data availability statement:

The datasets used during the current study are available from the corresponding author upon reasonable request.

## Sources of funding

This research received no external funding.

## Figures and Tables

**Table 1:** Baseline demographic and clinical characteristics of study participants

**Variable**	**Non-obese (n = 100)**	**Obese (n = 100)**	**p-value**
Age (years)	39.8 ± 8.2	40.5 ± 7.9	0.56
Sex (Male/Female)	54 / 46	57 / 43	0.68
BMI (kg/m^2^)	23.4 ± 1.8	32.6 ± 2.7	<0.001
Smoking (%)	18	22	0.47
Alcohol consumption (%)	12	15	0.52
Physical activity (%)	64	41	0.003
Diabetes (%)	14	28	0.01
Hypertension (%)	16	34	0.002
P < 0.05 was considered
statistically significant.

**Table 2:** Liver function biomarkers in obese vs. non-obese groups

**Parameter**	**Non-obese (Mean ± SD)**	**Obese (Mean ± SD)**	**p-value**
ALT (U/L)	21.32 ± 7.67	32.34 ± 11.55	0.001
AST (U/L)	20.58 ± 6.41	27.83 ± 9.13	0.02
ALP (U/L)	78.02 ± 16.80	102.38 ± 20.06	0.001
GGT (U/L)	25.62 ± 8.93	43.37 ± 13.33	<0.001
Albumin (g/dL)	4.06 ± 0.47	3.91 ± 0.66	<0.001
Prealbumin (mg/dL)	26.67 ± 3.46	28.25 ± 4.30	0.008
TBIL (µmol/L)	9.67 ± 2.67	7.23 ± 3.45	<0.001
DBIL (µmol/L)	3.45 ± 1.08	3.00 ± 1.11	<0.001
ALT = alanine aminotransferase;
AST = aspartate aminotransferase;
ALP = alkaline phosphatase;
GGT = gamma-glutamyl transferase;
TBIL = total bilirubin;
DBIL = direct bilirubin.
P < 0.05 was considered
statistically significant.

**Table 3:** Inflammatory biomarkers in obese vs. non-obese groups

**Parameter**	**Non-obese (Mean ± SD)**	**Obese (Mean ± SD)**	**p-value**
CRP (mg/L)	2.44 ± 2.82	9.33 ± 11.55	<0.001
IL-6 (pg/mL)	5.07 ± 2.26	8.10 ± 5.53	0.044
TNF-α (pg/mL)	5.36 ± 9.21	12.30 ± 20.14	0.04
IL-1 β (pg/mL)	0.75 ± 0.28	1.06 ± 0.42	0.144
Fibrinogen (g/L)	2.66 ± 0.42	3.39 ± 0.58	0.01
ESR (mm/hr)	11.3 ± 3.8	14.8 ± 5.3	0.09

**Table 4:** Vitamin D-related parameters and parathyroid hormone in obese vs. non-obese groups

**Parameter**	**Non-obese (Mean ± SD)**	**Obese (Mean ± SD)**	**p-value**
25(OH)D (ng/mL)	27.17 ± 11.00	26.71 ± 9.33	0.03
Bioavailable Vitamin D (ng/mL)	4.79 ± 1.13	4.88 ± 1.17	0.003
Free Vitamin D (pg/mL)	15.04 ± 3.01	15.79 ± 3.18	0.011
DBP (mg/L)	338 ± 56	413 ± 69	0.231
PTH (pg/mL)	38.5 ± 8.5	49.8 ± 10.6	0.01
Calcium (mg/dL)	8.74 ± 0.38	9.75 ± 0.53	0.3
25(OH)D = 25-hydroxyvitamin D;
DBP = vitamin D-binding protein;
PTH = parathyroid hormone.
P < 0.05 was considered
statistically significant.
P-values were calculated using independent t-tests.

**Table 5:** Multivariable logistic regression analysis of liver function biomarkers associated with obesity

**Variable**	**Adjusted OR (95% CI)**	**p-value**
ALT	1.22 (1.10-1.36)	0.001
AST	1.18 (1.02-1.32)	0.02
ALP	1.24 (1.11-1.38)	0.001
GGT	1.35 (1.20-1.52)	<0.001
Albumin	0.82 (0.71-0.94)	<0.001
Prealbumin	0.85 (0.76-0.96)	0.008
TBIL	0.94 (0.82-1.07)	0.31
DBIL	0.91 (0.79-1.05)	0.22
ALT = alanine aminotransferase;
AST = aspartate aminotransferase;
ALP = alkaline phosphatase;
GGT = gamma-glutamyl transferase;
TBIL = total bilirubin;
DBIL = direct bilirubin

**Table 6:** Multivariable logistic regression analysis of inflammatory biomarkers associated with obesity

**Variable**	**Adjusted OR (95% CI)**	**p-value**
CRP	1.38 (1.24 - 1.54)	<0.001
IL-6	1.21 (1.05 - 1.38)	0.044
TNF-α	1.09 (0.95 - 1.25)	0.18
IL-1 β	1.12 (0.97 - 1.29)	0.14
Fibrinogen	1.19 (1.07 - 1.32)	0.01
ESR	1.08 (0.96 - 1.21)	0.09

**Table 7:** Multivariable logistic regression analysis of vitamin D-related parameters and calcium associated with obesity

**Variable**	**Adjusted OR (95% CI)**	**p-value**
25(OH)D	0.79 (0.68 - 0.91)	0.03
Bioavailable Vitamin D	0.83 (0.72 - 0.95)	0.003
Free Vitamin D	0.85 (0.74 - 0.97)	0.011
DBP	1.08 (0.95 - 1.22)	0.23
PTH	1.18 (1.06 - 1.31)	0.01
Calcium	0.95 (0.81 - 1.11)	0.3
